# Integrated Lipidomics and Metabolomics Reveal Stage-Dependent Differences in Flavor Precursor Composition Between Higher- and Lower-Body-Weight Beijing-You Chickens

**DOI:** 10.3390/foods15091564

**Published:** 2026-05-01

**Authors:** Xia Chen, Jian Zhang, Xiaoyue Zhang, Cheng Chang, Hongchang Gu, Zhixun Yan, Lingchao Zeng, Ailian Geng, Jing Cao, Qin Chu, Huagui Liu

**Affiliations:** Institute of Animal Husbandry and Veterinary Medicine, Beijing Academy of Agriculture and Forestry Sciences, Beijing 100097, China; chenxia_91@163.com (X.C.); zjcau@126.com (J.Z.); 13240150468@163.com (X.Z.); changeng02@163.com (C.C.); guhc@cau.edu.cn (H.G.); yanzhixun2008@sina.com (Z.Y.); yfzjc@163.com (L.Z.); ailiangengcau@126.com (A.G.); caojing2046555@163.com (J.C.)

**Keywords:** chicken, body weight variation, developmental stage, flavor precursor, lipidomics, metabolomics

## Abstract

Body weight variation within a breed may be associated with meat flavor in chickens, but its relationship with flavor-related precursor composition across developmental stages remains unclear. Here, integrated lipidomics and metabolomics were applied to compare breast muscle from Beijing-You chickens sampled from the same cohort at 90, 110, 130, and 150 d in a stage-wise design. At each stage, higher-body-weight (HBW) and lower-body-weight (LBW) groups were independently defined from the upper and lower tails of the body weight distribution at that age. A total of 440, 259, 161, and 324 differential lipids, as well as 491, 257, 291, and 402 differential metabolites, were identified at the four stages, respectively. However, only 23 lipids and 3 metabolites were shared across all stages, indicating that metabolic differences between the HBW and LBW groups varied markedly across developmental stages. Differential lipids were mainly distributed among phosphatidylcholine, phosphatidylethanolamine, and phosphatidylserine, while glycerophospholipid metabolism was consistently identified in both lipidomic and metabolomic analyses. Notably, a key transition was observed between 110 and 130 d, during which the predominant direction of PUFA-like differential lipids shifted from HBW to LBW predominance. Representative differential metabolites included N-acetyl-L-methionine, N-methyl-L-glutamic acid, and γ-glutamyl-5-hydroxytryptophan, suggesting alterations in amino acid- and peptide-related metabolism. Overall, these findings provide insight into stage-dependent variation in flavor-related precursor composition within a breed across developmental stages. However, their direct contribution to flavor remains to be validated.

## 1. Introduction

Flavor is an important quality attribute of poultry meat that strongly influences consumer preference. It arises from a complex interplay of water- and fat-soluble precursors in meat during cooking [1]. Water-soluble precursors, including amino acids, small peptides, nucleotides, phosphorylated sugars, and thiamine, contribute to taste attributes such as umami, sweetness, and bitterness and also participate in Maillard-type reactions during heating [2,3]. Fat-soluble precursors mainly include phospholipids, triglycerides, fatty acids, and sterol esters, among which phospholipids are particularly important because they are enriched in unsaturated fatty acids that readily undergo thermal oxidation [1,4]. Interactions between Maillard-type reactions and lipid oxidation further generate a broad range of odor-active volatiles, including aldehydes, ketones, and sulfur-containing compounds, that shape the characteristic aroma of cooked chicken. Therefore, both water-soluble and lipid-soluble precursors should be considered when investigating the biochemical basis of meat flavor.

Eating quality is an important determinant of chicken meat acceptance and purchasing behavior. In China, consumer studies have shown that taste preference significantly influences chicken purchasing decisions [5]. For example, Wang et al. identified breed-specific aroma compounds in native Chinese chickens that were absent in white-feathered broilers [6], while Deng et al. showed that Chinese yellow-feathered chickens exhibited breed-specific flavor characteristics and that relatively younger birds achieved higher overall sensory scores [7]. These findings have increased interest in understanding how body weight variation within and between chicken populations is associated with meat quality and flavor. Previous studies have suggested that the composition of flavor-related precursors differs markedly between fast-growing (FG) and slow-growing (SG) chickens. For example, Xiao et al. [8] reported that slow-growing chickens, including Wuding chicken and Yanjin Silky Fowl, contained higher levels of water-soluble small molecules and fatty acids than fast-growing Cobb broilers. Similarly, Chen et al. [9] found that lipids and amino acids were the major classes of differential flavor precursors between FG broilers and SG Beijing-You chickens (BYC). Zhao et al. [10] further showed that, although triglyceride content did not differ significantly between Arbor Acres broilers and Beijing-You chickens, phospholipid concentrations were 2.7-fold higher in Beijing-You chickens. However, despite these findings, an important knowledge gap remains. Most previous omics-based studies of chicken meat quality have relied on comparisons between breeds or genetic lines, making it difficult to distinguish growth rate-associated metabolic differences from breed-background effects. Therefore, it remains unclear whether comparable metabolic divergence can also be detected between phenotypic extremes within a breed, in the absence of major between-breed genetic differences.

Moreover, growth rate-associated metabolic differences are unlikely to be static throughout development, because muscle metabolism and flavor precursor accumulation are both highly age-dependent. Although previous omics studies comparing FG and SG chickens have provided useful snapshots of metabolic differences, these studies were generally conducted at a single developmental stage or market age [9]. By contrast, age-dependent changes in chicken muscle metabolites and meat quality traits have been reported across development [11,12,13]. Therefore, it remains unclear whether such metabolic divergence is stable throughout growth or varies across developmental stages.

Beijing-You chicken (BYC) is a traditional Chinese breed known for its distinctive meat quality and typically reaches market age at around 120 d. Importantly, BYC populations show substantial variation in body weight and growth performance [14], while birds within the same breed retain a more comparable genetic background than those used in between-breed comparisons. This makes BYC a useful within-breed model for investigating flavor-related metabolic differences associated with phenotypic variation in body weight and growth performance while minimizing confounding from breed background. Notably, Yang et al. (2025) reported no significant differences in carcass traits, meat quality parameters, or intramuscular fat content between different body weight groups of BYC [15]. However, preliminary electronic nose and electronic tongue analyses suggested differences in odor and taste profiles between the two groups, implying that within-breed variation in body weight status at a given developmental stage may still be associated with differences in flavor-related metabolic composition. Taken together, these observations indicate that a within-breed, multi-stage design is needed to disentangle within-breed phenotypic metabolic variation from breed effects and to determine whether such variation is stage-dependent across development.

In the present study, we used an integrated lipidomics–metabolomics approach to investigate stage-dependent variation between body weight-defined phenotypic groups in flavor precursor composition in the breast muscle of higher-body-weight (HBW) and lower-body-weight (LBW) Beijing-You chickens sampled at 90, 110, 130, and 150 d of age. Specifically, we aimed to characterize the major metabolic differences between the HBW and LBW groups within a breed, identify the lipid and metabolite classes most closely associated with this divergence, and determine whether this metabolic divergence exhibits developmental stage dependence, including the key developmental window during which the differences are most pronounced.

## 2. Materials and Methods

### 2.1. Animals and Samples

A cohort of 200 female Beijing-You chickens was housed at the Beijing-You Chicken Resource Conservation Farm (Beijing, China). Only female birds were included in order to minimize sex-related variation and improve biological comparability among body weight-defined groups [16,17], because sex is known to influence poultry meat traits and chicken breast muscle molecular phenotypes. All birds originated from the same cohort and were reared under identical housing, feeding, and management conditions throughout the study period. Birds were kept in individual cages and provided with the same corn–soybean-based diet and water ad libitum.

Body weight was recorded at 90, 110, 130, and 150 d of age. Beijing-You chicken is a Chinese indigenous breed, and substantial within-population variation in growth performance can be observed in this population. Therefore, at each developmental stage, birds from the upper and lower tails of the body weight distribution were classified as higher-body-weight (HBW) and lower-body-weight (LBW) groups, respectively. These groups do not represent genetically distinct fast- and slow-growing lines but stage-specific phenotypic groups defined within the same Beijing-You chicken cohort. In this study, the HBW and LBW groups differed significantly in body weight at each developmental stage (*p* < 0.01, Table S1). Because breast muscle samples were obtained by terminal slaughter, birds were independently selected at each developmental stage from the same cohort for subsequent analyses. Ten birds per group were slaughtered at each age. After a 12-h fast, birds were slaughtered by carotid bleeding, and breast muscle samples were collected, immediately flash-frozen in liquid nitrogen, transported on dry ice, and stored at −80 °C until lipidomic and metabolomic analyses.

### 2.2. Lipidomic Analysis

Lipids were extracted from breast muscle samples as follows. Briefly, 50 mg tissue was mixed with 280 μL methanol:water (2:5, *v*/*v*) and 400 μL methyl tert-butyl ether (MTBE), and then homogenized at −10°C using a tissue crusher (Wonbio-96c, Shanghai wonbio technology Co., Ltd., Shanghai, China) at 50 Hz for 6 min. The homogenate was sonicated at 40 kHz for 30 min at 5 °C, incubated at −20 °C for 30 min, and centrifuged (13,000× *g*, 15 min, 4 °C). The upper lipid-containing phase (350 μL) was collected, evaporated under nitrogen, and reconstituted in 100 μL isopropanol:acetonitrile (1:1, *v*/*v*) with brief sonication at 5 °C. After centrifugation at 13,000× *g* for 15 min at 4 °C, and the supernatant was transferred to sample vials. A 5 μL aliquot was injected into the UHPLC-MS/MS system. A pooled quality control (QC) sample was prepared by combining equal volumes of all experimental samples to test system stability.

Lipid profiling was conducted using a UHPLC-Q Exactive HF-X system (Thermo Fisher Scientific, Waltham, MA, USA) equipped with an Accucore C30 column (100 mm × 2.1 mm i.d., 2.6 μm; Thermo Fisher, USA). The mobile phases consisted of (A) 10 mM ammonium acetate in acetonitrile:water (1:1, *v*/*v*) with 0.1% formic acid and (B) 2 mM ammonium acetate in acetonitrile:isopropanol:water (10:88:2, *v*/*v*/*v*) containing 0.02% formic acid. The column temperature was maintained at 40 °C. The gradient elution program was as follows: 35–60% B for 0–4 min, 60–85% B for 4–12 min, 85–100% B for 12–15 min, held at 100% B for 15–17 min, returned to 35% B during 17–18 min, and held at 35% B for 18–20 min. Mass spectrometric detection was performed using a heated electrospray ionization source in positive and negative ion modes (±3000 V). Data were acquired in data-dependent acquisition modes across an *m*/*z* range of 200–2000.

### 2.3. Metabolomic Analysis

Fifty milligrams of tissue was homogenized with a 6 mm stainless steel bead in 400 μL of methanol/water (4:1, *v*/*v*) containing 20 μg/mL L-2-chlorophenylalanine (internal standard) using a cryogenic tissue grinder (Wonbio-96c, Shanghai wonbio technology Co., Ltd., Shanghai, China) at −10 °C (50 Hz, 6 min). Ultrasonic extraction was subsequently performed at 40 kHz for 30 min at 5 °C, incubated at −20 °C for 30 min, and centrifuged at 13,000× *g*, for 15 min at 4 °C. The supernatant was collected for LC-MS/MS analysis. A pooled quality control (QC) sample was prepared by mixing equal aliquots of supernatants from all samples.

Metabolite profiling was performed using a UHPLC system coupled to an Orbitrap Exploris 240 mass spectrometer (Thermo Fisher Scientific, Waltham, MA, USA) equipped with an ACQUITY UPLC HSS T3 column (100 mm × 2.1 mm, 1.8 μm; Waters, Milford, USA). The mobile phases consisted of A: water containing 0.1% formic acid and 5% acetonitrile and B: acetonitrile/isopropanol/water (47.5:47.5:5, *v*/*v*/*v*) containing 0.1% formic acid. The injection volume was 3 μL, and the column temperature was maintained at 40 °C. Mass spectrometric data were acquired in both positive and negative electrospray ionization modes using Full MS/dd-MS^2^ acquisition. The scan range was *m*/*z* 70–1050. The sheath gas flow rate was 60 arb, and the auxiliary gas flow rate was 20 arb. The heater temperature and capillary temperature were set to 350 °C and 320 °C, respectively. The spray voltage was 3.4 kV in positive ion mode and −3.0 kV in negative ion mode. The S-Lens RF level was set to 70, and the normalized collision energy was set to 20, 40, and 60 eV. The resolution was 60,000 for Full MS and 15,000 for MS^2^.

### 2.4. Data Processing and Statistical Analysis

Raw lipidomics data were processed using LipidSearch 4.1 (Thermo Fisher Scientific) for peak extraction, alignment, and lipid identification. Lipid features detected in at least 80% of samples within either the HBW or LBW group at a given developmental stage were retained. Missing values were imputed using the minimum observed value for the corresponding feature. The data were sum-normalized and log10-transformed, and lipid features with a relative standard deviation (RSD) >30% in pooled QC samples were excluded from subsequent analyses.

Raw metabolomics data were processed using Progenesis QI (Waters Corporation, Milford, CT, USA) for peak picking, alignment, deconvolution, and generation of a feature intensity matrix. Signals derived from internal standards, background noise, and column bleed were removed before downstream analysis. Metabolite annotation was performed against the HMDB, METLIN, and the Majorbio cloud platform database. Only metabolite features present in at least 80% of samples within either the HBW or LBW group at each developmental stage were retained for further analysis.

All statistical analyses were performed separately for the four developmental stages (90, 110, 130, and 150 d). Orthogonal partial least squares-discriminant analysis (OPLS-DA) was conducted using the ropls package in R (version 4.2.0) to evaluate global separation between the HBW and LBW groups in the lipidomic and metabolomic datasets. Model robustness was assessed by permutation testing. Permutation testing was used as an additional assessment of the supervised models. OPLS-DA was used here primarily as a supervised exploratory tool for stage-specific group discrimination and feature prioritization. Differential lipids (DLs) and differential metabolites (DMs) were identified based on variable importance in projection (VIP) > 1 in the OPLS-DA model and *p* < 0.05 from Benjamini–Hochberg false discovery rate (FDR).

For lipidomic analyses, to characterize lipid changes related to unsaturation, differential lipids containing at least one polyunsaturated fatty acyl chain were extracted and designated as PUFA-like differential lipids (PUFA-like DLs). To characterize stage-related response patterns of lipid differences, DLs were classified according to their occurrence across the four sampling time points into five categories: the early-stage pattern (detected only at 90 d), the middle-stage pattern (detected only at 110 and/or 130 d), the late-stage pattern (detected only at 150 d), the persistent pattern (detected at all four stages), and other patterns (all remaining discontinuous combinations).

For cross-omics integration, lipidomic and metabolomic data were first analyzed in parallel at each developmental stage to identify recurrent patterns shared across the two platforms, including stage dependence, recurrent pathway-level signals, and representative molecular classes. A targeted integration analysis was then performed by correlating the 23 differential lipids shared across the four developmental stages with representative differential metabolites, including the metabolites shared across stages and the top VIP-ranked metabolites identified at different developmental stages. Pearson correlation analysis and correlation network visualization were used to characterize coordinated lipid–metabolite variation. Thus, the present integration strategy was based on parallel cross-platform interpretation and targeted correlation/network analysis, rather than on direct merging of raw features from the two datasets.

## 3. Results

### 3.1. Stage-Dependent Lipidomic Differences Between Higher- and Lower-Body-Weight Groups

To characterize lipidomic differences associated with body weight status at each developmental stage, breast muscle lipid profiles of HBW and LBW chickens were compared at 90, 110, 130, and 150 days of age. OPLS-DA showed separation between the two groups at each developmental stage in both positive and negative ion modes (Figure S1), supporting stage-specific discrimination at the exploratory level. Permutation analyses were performed as an additional assessment of the supervised models, with all R^2^Y values exceeding 0.95 and Q^2^ values above 0.50 (Figure S2). These results support the utility of the models for exploratory discrimination and feature prioritization.

**Figure 1:**
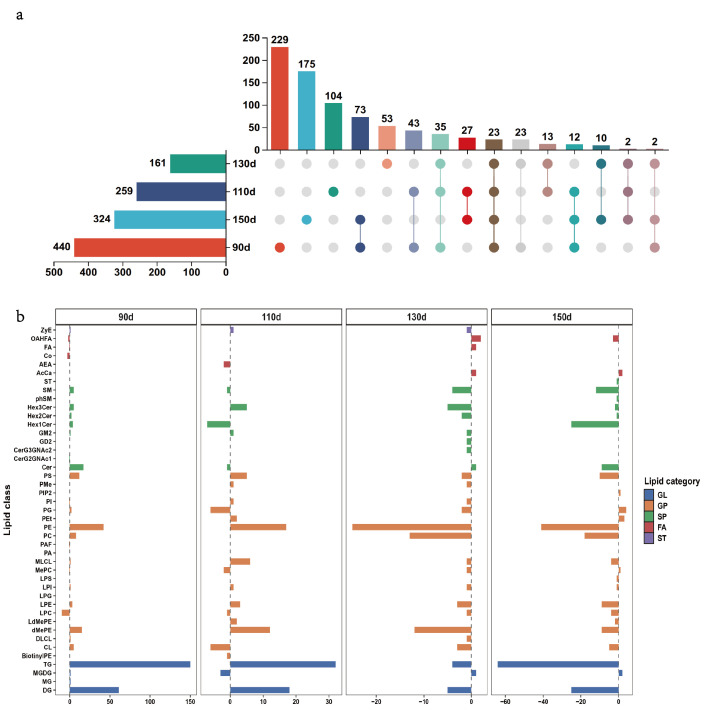
Overlap and class distribution of differential lipids between higher- and lower-body-weight Beijing-You chickens across developmental stages. (**a**) UpSet plot showing the overlap of differential lipids (DLs) identified in HBW vs. LBW comparisons across the four stages. (**b**) Lipid category/class distribution of DLs at each stage, grouped into glycerolipids (GLs), glycerophospholipids (GPs), sphingolipids (SPs), fatty acyls (FAs), and sterol lipids (STs). Different colors in panel (**a**) distinguish stage-specific and overlapping lipid sets; colors in panel (**b**) indicate lipid categories.

A total of 440, 259, 161, and 324 differential lipids (DLs) were identified in the HBW vs. LBW comparisons at 90, 110, 130, and 150 d, respectively (Figure 1a, Table S2). UpSet analysis further showed that 229, 104, 73, and 175 DLs were specific to the four corresponding developmental stages, whereas only 23 DLs were shared across all four stages (Figure 1a). Lipid class annotation showed that DLs were mainly distributed among several major classes, including phosphatidylcholine (PC), phosphatidylethanolamine (PE), phosphatidylserine (PS), triglyceride (TG), and diglyceride (DG) (Figure 1b). Notably, a clear shift in the direction of lipid regulation was observed between 110 and 130 d, with several major lipid classes shifting from predominantly higher in HBW chickens at 90–110 d to predominantly higher in LBW chickens at 130–150 d. These results indicate that most lipid differences between the HBW and LBW groups were highly stage-dependent.

**Figure 2:**
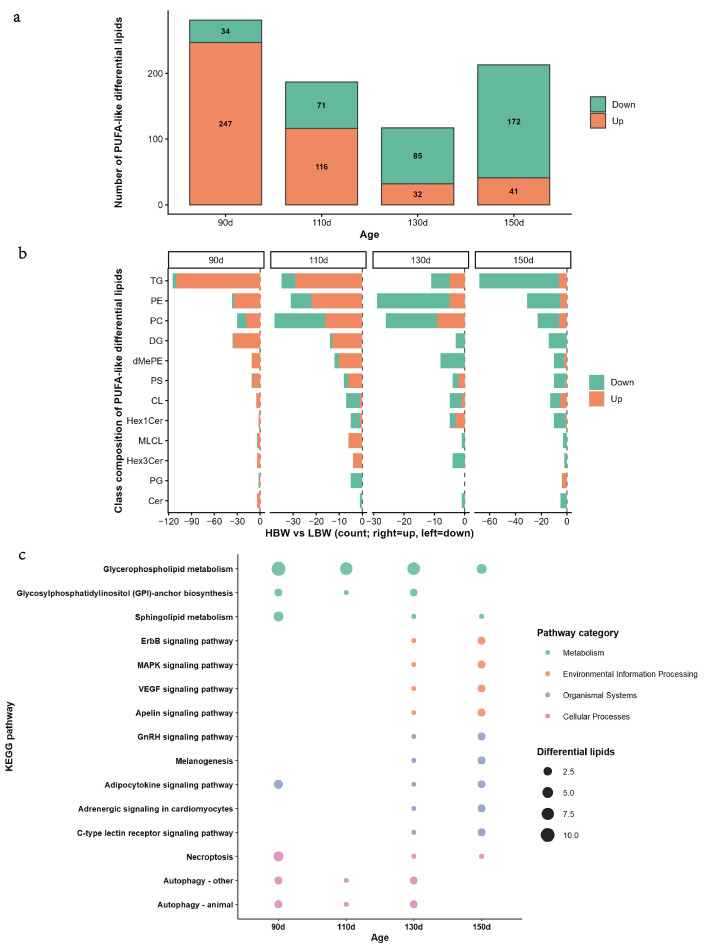
Stage-dependent patterns of PUFA-like differential lipids between higher-body-weight (HBW) and lower-body-weight (LBW) Beijing-You chickens. (**a**) Numbers of PUFA-like DLs (differential lipids containing at least one polyunsaturated fatty acyl chain) enriched in HBW or LBW chickens at each stage. (**b**) Class composition of PUFA-like DLs at each stage. Bars represent the counts of PUFA-like DLs within each lipid class, with positive values indicating HBW-enriched and negative values indicating LBW-enriched. (**c**) KEGG pathway annotation of DLs identified in HBW vs LBW comparisons at each stage.

Given that lipid unsaturation is closely related to lipid-derived flavor precursors, the distribution of PUFA-like DLs, defined here as differential lipids containing at least one polyunsaturated fatty acyl chain, was further summarized in Figure 2a. At 90 and 110 d, PUFA-like DLs with higher abundance in HBW than in LBW predominated (247 vs. 34 at 90 d; 116 vs. 71 at 110 d). In contrast, at 130 and 150 d, PUFA-like DLs with lower abundance in HBW than in LBW became predominant (32 vs. 85 at 130 d; 41 vs. 172 at 150 d), indicating a directional shift in the relative abundance pattern of PUFA-like DLs between 110 and 130 d. To further determine which lipid classes contributed to this transition, the lipid class composition of PUFA-like DLs was analyzed (Figure 2b). PUFA-like DLs were primarily distributed among TG, PE, PC, and DG across stages. The number and direction of PUFA-like DLs within each lipid class differed among stages, and these class-level changes were consistent with the pattern observed.

To explore the potential metabolic pathways underlying these lipid alterations, DLs were further mapped to KEGG pathways (Figure 2c). DLs were involved in multiple pathways, including glycerophospholipid metabolism, glycosylphosphatidylinositol (GPI)-anchor biosynthesis, and sphingolipid metabolism, as well as additional signaling- and cellular process-related pathways. Among these pathways, glycerophospholipid metabolism was consistently enriched at all four stages, indicating that it was a key pathway underlying lipid remodeling between the HBW and LBW groups during development.

Together, these results showed that lipidomic differences between HBW and LBW groups were highly stage-dependent. Notably, the interval between 110 and 130 d was supported as a transition window by multiple convergent observations, including the reversal in the predominant direction of PUFA-like differential lipids, the corresponding shift in the major contributing lipid classes, and the stage-related redistribution of differential lipids from earlier TG-dominant patterns toward later phospholipid-associated patterns.

### 3.2. Stage-Dependent Response Patterns of Differential Lipids

To further characterize how specific lipids contributed to the stage-related differences between HBW and LBW groups, DLs were classified into distinct stage-related response patterns.

**Figure 3:**
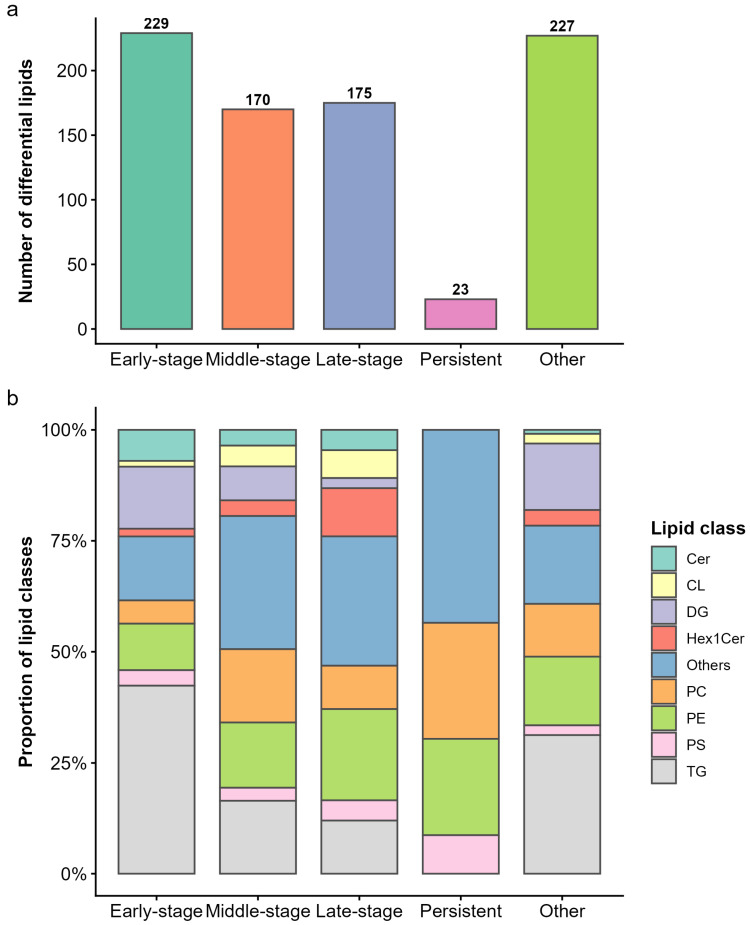
Stage-dependent response patterns of differential lipids. (**a**) Differential lipids were grouped into distinct stage-related response patterns based on their occurrence across growth stages, including early-stage (90 d), middle-stage (110 and 130 d), late-stage (150 d), persistent (detected at all four stages), and other patterns (irregular or discontinuous occurrence across stages). Bars indicate the number of DLs assigned to each pattern. (**b**) Lipid class composition of DLs within each stage-related response pattern. The stacked bars represent the relative proportion of different lipid classes in each pattern.

As shown in Figure 3a, most DLs exhibited stage-restricted response patterns, with a large proportion assigned to early-stage (229 lipids), middle-stage (170 lipids), and late-stage (175 lipids) categories. In addition, 23 DLs were present at all four stages and were therefore classified as the persistent pattern. To determine whether these stage-related response patterns were associated with distinct lipid classes, the lipid class composition of DLs was further examined (Figure 3b). Early-stage DLs were predominantly composed of GL, with TG accounting for the largest proportion, whereas GP classes, particularly PE and PC, accounted for a smaller proportion. In contrast, middle-stage and late-stage DLs showed a reduced proportion of TG and a greater contribution of GP-related classes. Notably, persistent DLs were mainly composed of GP, with PC and PE representing the major lipid classes and only a minor contribution from TG. Overall, these results showed a progressive shift in lipid class composition from TG-enriched patterns at the early stage to GP-enriched profiles in later and persistent patterns.

**Figure 4:**
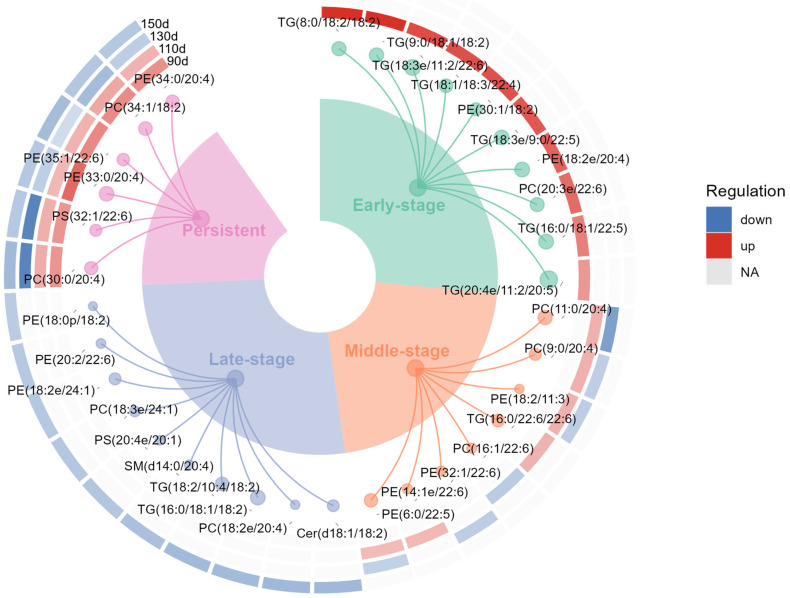
Circular plot illustrating stage-dependent differential abundance patterns of representative DLs across growth stages. Colored sectors indicate different stage-related patterns, and links represent the presence and direction of differential abundance (HBW vs. LBW) of individual lipids at each stage. Red indicates significantly up-regulated lipids, blue indicates significantly down-regulated lipids, and grey indicates non-significant changes. The color intensity reflects the magnitude of the VIP value.

To further illustrate representative lipidomic changes underlying different stage-related response patterns, representative DLs were selected based on *p* value and fold change between HBW and LBW groups. As shown in Figure 4, 10 early-stage, 8 middle-stage, 10 late-stage, and 6 persistent lipids were identified. Early-stage representative lipids were mainly TG species, such as TG(20:4e/11:2/20:5), TG(18:3e/11:2/22:6), and TG(16:0/18:1/22:5), most of which showed higher abundance in HBW chickens at 90 d. In contrast, middle-stage representative lipids were mainly GP species, particularly several PC and PE molecules, whereas late-stage representative lipids included PE, PC, TG, and several complex lipids and were consistently lower in HBW chickens at 150 d. Persistent representative lipids were predominantly phospholipid species, including PC, PE, and PS, and typically showed higher abundance in HBW birds at 90–110 d but lower abundance at 130–150 d. Collectively, these representative lipids indicate that the stage-related difference between HBW and LBW chickens involved not only shifts in dominant lipid classes, but also pattern-specific changes in the direction and timing of lipid abundance.

### 3.3. Body Weight-Associated Metabolomic Alterations Across Developmental Stages

To further investigate metabolic differences associated with body weight status at each developmental stage, untargeted metabolomic profiling was conducted to compare HBW and LBW groups at four developmental stages. The OPLS-DA plots showed separation between HBW and LBW groups at each age group (Figure S3), consistent with stage-specific metabolomic differences at the exploratory level. Permutation tests of the OPLS-DA models yielded all R2Y above 0.99 and Q2 greater than 0.7 (Figure S3c). These results support the use of OPLS-DA for exploratory discrimination.

**Figure 5:**
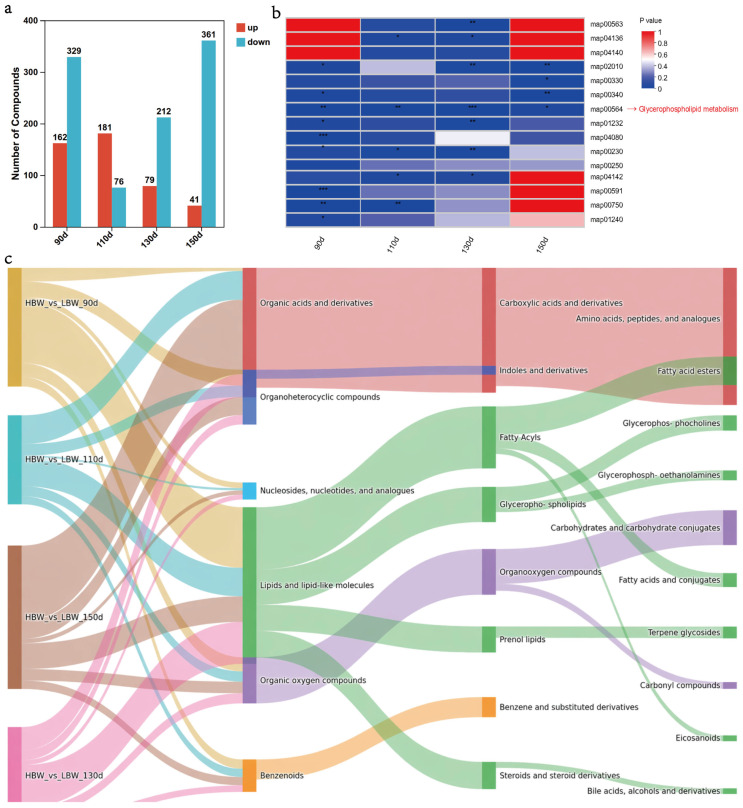
Differential metabolite profiling between higher-body-weight (HBW) and lower-body-weight (LBW) chickens at 90, 110, 130, and 150 d of age. (**a**) Bar plot showing the differential metabolites identified between HBW and LBW groups at four developmental stages. (**b**) Sankey plot illustrates the hierarchical classification of differential metabolites across four age comparisons. (**c**) KEGG pathway enrichment heatmap of differential metabolites at each age. *, **, and *** indicate *p* < 0.05, *p* < 0.01, and *p* < 0.001, respectively.

Marked stage-dependent variation in the number and direction of differential abundance of DMs was observed across the four developmental stages (Figure 5a, Table S3). A total of 491, 257, 291, and 402 DMs were identified at 90, 110, 130, and 150 d, respectively. Among these, 162, 181, 79, and 41 metabolites exhibited higher abundance in HBW chickens, whereas 329, 76, 212, and 361 metabolites showed lower abundance relative to LBW chickens at corresponding stages. Overall, DMs with lower abundance in HBW chickens predominated at 90, 130, and 150 d, whereas metabolites with higher abundance were relatively more prevalent at 110 d.

To characterize the chemical composition of these DMs, metabolites were annotated according to the HMDB classification (Figure 5b). Across all stages, DMs were primarily assigned to the superclass of organic acids and derivatives, followed by lipids and lipid-like molecules and organic oxygen compounds. At the class level, carboxylic acids and derivatives represented the dominant metabolite class, corresponding mainly to amino acids, peptides, and analogues. In addition, lipid-related classes, including fatty acyls and glycerophospholipids, were represented among the DMs. These results indicate that both amino acid- and lipid-associated metabolites were represented among the differences observed between the HBW and LBW groups.

KEGG pathway enrichment analysis was subsequently performed to identify metabolic pathways associated with the detected DMs (Figure 5c). KEGG pathway enrichment analysis showed stage-dependent variation across development, but glycerophospholipid metabolism remained the most recurrent pathway-level signal in the metabolomic data (Figure 5c). This result, together with the HMDB classification, further suggests that glycerophospholipid remodeling is a prominent metabolic feature underlying the differences between the HBW and LBW groups during development.

### 3.4. Identification of Representative Differential Metabolites Associated with Body Weight-Defined Group Differences

**Figure 6:**
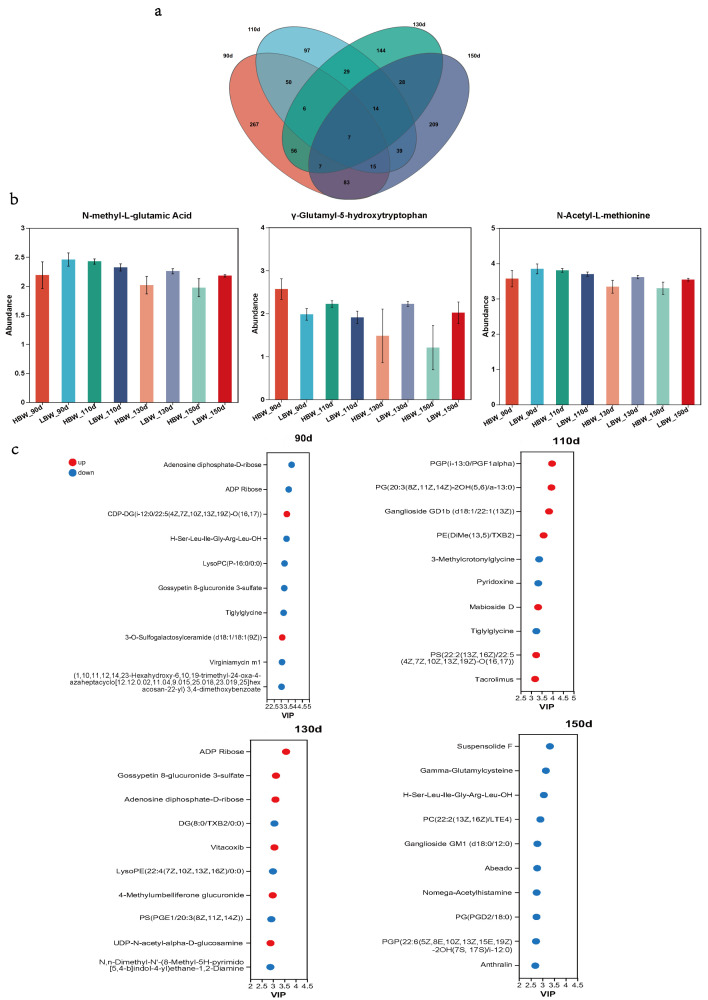
Identification of representative metabolites associated with body weight-defined group differences in Beijing-You chicken breast muscle. (**a**) Venn diagram showing the overlap of differential metabolites between higher-body-weight (HBW) and lower-body-weight (LBW) groups across four developmental stages (90, 110, 130, and 150 d). (**b**) Abundance patterns of the three differential metabolites shared across all developmental stages. (**c**) Top 10 metabolites contributing to the separation between HBW and LBW groups at each developmental stage based on variable importance in projection (VIP) values from the OPLS-DA model.

To further identify representative metabolites associated with the metabolic difference between HBW and LBW groups, differential metabolites detected at the four developmental stages were compared. As shown in Figure 6a, most differential metabolites were stage-specific, whereas only three metabolites were shared across all four developmental stages, indicating that metabolomic divergence between the HBW and LBW groups was largely stage-dependent. Therefore, subsequent interpretation focused primarily on these shared metabolites and the top discriminative metabolites identified at each stage.

The abundance patterns of the three shared metabolites are shown in Figure 6b. These metabolites included γ-Glutamyl-5-hydroxytryptophan, N-methyl-L-glutamic acid, and N-acetyl-L-methionine. Among them, N-acetyl-L-methionine and N-methyl-L-glutamic acid generally showed higher abundance in LBW chickens across multiple stages. In contrast, γ-Glutamyl-5-hydroxytryptophan showed greater temporal fluctuation, with more pronounced group differences at 130 and 150 d, particularly a higher abundance in LBW chickens at 150 d.

To identify metabolites contributing strongly to group discrimination, metabolites with high VIP values were screened for each developmental stage (Figure 6c). Most top-ranked metabolites were stage-specific, although a few were repeatedly detected across stages. For example, ADP-ribose and adenosine diphosphate-D-ribose were identified at both 90 and 130 d. In addition, several lipid-related metabolites, including PG- and PC-related molecules, were detected among the top discriminative features at different stages, indicating that lipid-related metabolites also contributed to the metabolic difference between HBW and LBW groups during development.

### 3.5. Correlation Networks Between Differential Lipids and Metabolites

To further characterize coordinated molecular features underlying the metabolic differences between HBW and LBW groups, an integrative analysis was conducted using the 23 differential lipids shared across developmental stages and representative differential metabolites. The latter included the three metabolites shared across all four stages and the top VIP-ranked metabolites identified at different developmental stages.

**Figure 7:**
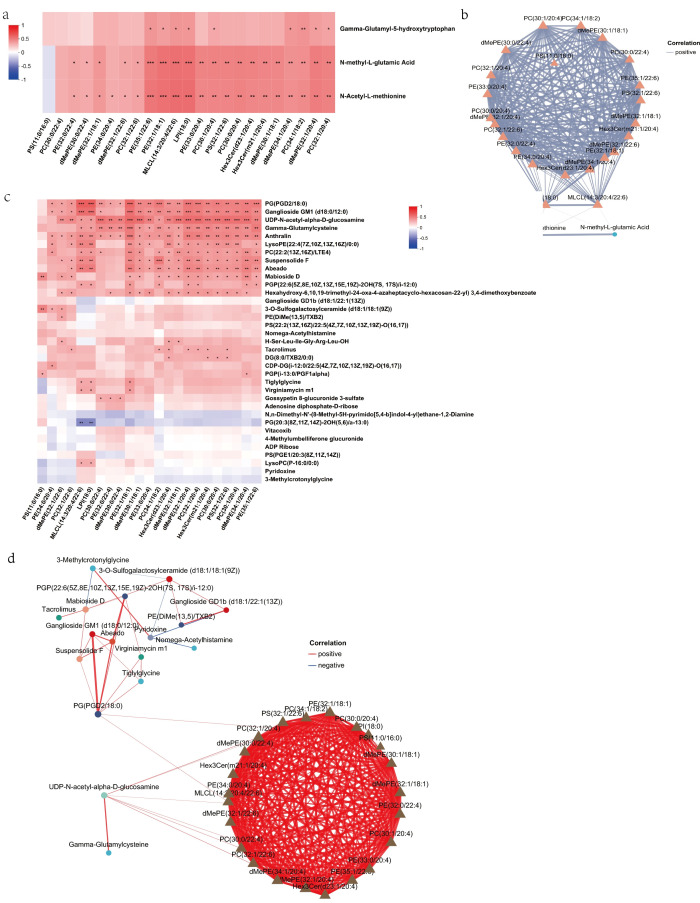
The correlation analysis of lipidomics and metabolomics. (**a**) Correlation analysis of the 23 overlapping differential lipids (DLs) and 3 overlapping differential metabolites (DMs). (**b**) Correlation network analysis of 23 overlapping DLs and 3 overlapping DMs with Pearson correlation coefficient r > 0.6. (**c**) Pearson correlation analysis of the 23 overlapping DLs and the top VIP-ranked DMs. (**d**) Correlation network analysis between the 23 overlapping DLs and the top VIP-ranked DMs with r > 0.6. *, **, and *** indicate *p* < 0.05, *p* < 0.01, and *p* < 0.001, respectively.

Pearson correlation analysis showed that N-methyl-L-glutamic acid and N-acetyl-L-methionine exhibited extensive positive correlations with the overlapping DLs, whereas γ-Glutamyl-5-hydroxytryptophan showed comparatively fewer significant associations (Figure 7a). In the correlation network constructed using r > 0.6, N-methyl-L-glutamic acid and N-acetyl-L-methionine were connected with multiple lipid species, including LPI(18:0), MLCL(14:3/20:4/22:6), PC-, PE-, PS-, and dMePE-related lipids (Figure 7b), indicating coordinated variation between these metabolites and phospholipid-associated lipid species.

Further correlation analysis between the 23 overlapping DLs and the top 10 VIP-ranked DMs identified at different developmental stages revealed extensive lipid-metabolite associations (Figure 7c). Several metabolites, including UDP-N-acetyl-α-D-glucosamine and γ-glutamylcysteine, showed strong correlations with multiple lipid species. Network analysis further indicated that UDP-N-acetyl-α-D-glucosamine was closely associated with a cluster of glycerophospholipid-related DLs, including MLCL(14:3/20:4/22:6), LPI(18:0), dMePE(32:1/20:4), PC(32:1/20:4), and PE(32:0/22:4), whereas γ-glutamylcysteine was linked to a smaller subset of lipids. In addition, several metabolites formed a more compact subnetwork with PG(PGD2/18:0) and related lipid species, showing that different metabolites were associated with different lipid clusters within the integrated network.

Together, these results indicate that metabolic differences between the HBW and LBW groups were accompanied by coordinated associations between glycerophospholipid-related lipids and small-molecule metabolites within the integrated analysis.

## 4. Discussion

### 4.1. Body Weight-Defined Group Metabolic Differences Vary Markedly Across Developmental Stages

The OPLS-DA results are interpreted here primarily as exploratory evidence for group discrimination and feature prioritization, whereas the biological interpretation relies more on cross-stage consistency, recurrent pathway-level signals, and targeted cross-omics correlation patterns. In the present study, only 23 lipids and 3 metabolites remained significantly different between HBW and LBW chickens across all four developmental stages. This limited overlap indicates that metabolic differences between these body weight-defined phenotypic groups were largely stage-dependent rather than maintained as a stable signature throughout growth. Because the present study used stage-wise independent sampling from the same cohort rather than longitudinal tracking of fixed individuals, the observed differences are interpreted here as within-cohort comparisons across developmental stages.

This interpretation is consistent with previous studies showing that both growth-related differences and age-related physiological changes can reshape muscle metabolism in chickens. For example, metabolomic comparison of slow- and fast-growing chickens in Thailand revealed clear differences in flavor-associated metabolites, including amino acids, ATP-related compounds, and sugars, between growth types [18]. Likewise, age comparison in Lueyang black-bone chickens showed that slaughter age significantly affected metabolites, phospholipids, and volatile compounds, indicating that flavor-related biochemical profiles in poultry are strongly stage-dependent [13].

This pattern may reflect the combined influence of rapid growth and progressive tissue maturation. It was reported that chicken muscle metabolism changes markedly across developmental stages, with age-related shifts in lipid deposition, energy metabolism, and flavor-related metabolites [19,20]. Therefore, metabolic differences between HBW and LBW groups may reflect differences in the timing and extent of metabolic remodeling, rather than a fixed set of metabolite changes maintained throughout growth.

Taken together, these findings suggest that metabolic divergence between HBW and LBW groups within BYC is not constant throughout growth but varies substantially across developmental stages.

### 4.2. Phospholipid Remodeling Represents the Most Prominent Signal Linked to Lipid-Derived Flavor Precursor Variation

The overlapping DLs were dominated by phospholipid-related species, especially PC, PE, and PS, while glycerophospholipid metabolism was consistently highlighted by both lipidomic and metabolomic analyses. In parallel, the integrated correlation network revealed a lipid-centered co-variation pattern rather than isolated molecular changes. Together, these results suggest that membrane phospholipid remodeling is the most prominent metabolic feature associated with the divergence between HBW and LBW groups. This observation is mechanistically relevant to flavor precursor metabolism because membrane phospholipids are major reservoirs of polyunsaturated fatty acyl chains, which are highly susceptible to oxidation during thermal processing and can contribute to the formation of odor-active volatile compounds.

Compared with neutral lipids such as triacylglycerols, membrane phospholipids contain higher proportions of polyunsaturated fatty acids such as linoleic acid (C18:2) and arachidonic acid (C20:4) [21,22]. These unsaturated fatty acids are important substrates for lipid oxidation during cooking and contribute to the formation of volatile compounds such as hexanal and 2,4-decadienal, which are widely recognized as major contributors to roasted and meaty aromas [23,24]. In addition, lipid oxidation is also closely interconnected with Maillard-type reactions, and lipid-derived oxidation products may further interact with water-soluble intermediates to influence volatile formation during heating [24]. Therefore, the observed phospholipid remodeling in HBW and LBW groups is likely to affect the composition of lipid-derived precursor pools relevant to meat flavor. This interpretation is consistent with recent studies emphasizing the central role of phospholipid classes in chicken meat flavor chemistry and the value of lipidomics for identifying flavor-relevant lipid species in poultry muscle [25,26].

Beyond their relevance to lipid-derived flavor precursors, phospholipids are also central structural components of cellular membranes. The predominance of phospholipid-related signals suggests that the observed metabolic divergence may be particularly evident in membrane lipid composition. Phospholipids are major structural components of cellular and organelle membranes and are closely linked to membrane organization, remodeling, and fatty acyl composition [27,28]. Accordingly, differences in phospholipid classes such as PC and PE may indicate variation in membrane-related lipid remodeling during muscle development. Previous studies have shown that glycerophospholipids are among the dominant lipid classes associated with age-related and quality-related variation in chicken muscle [29]. Together, the present results indicate that coordinated phospholipid remodeling is a prominent feature of metabolic divergence between the HBW and LBW groups.

Another notable feature was that the differential molecules were not distributed as isolated changes, but instead formed a coordinated lipid-centered co-variation pattern. The correlation heatmap and network analysis indicated relatively dense connections among PC, PE, PS, dMePE, Hex3Cer, MLCL, and LPI species, many of which contained unsaturated fatty acyl chains such as 20:4, 22:6, or 18:2. This coordinated pattern suggests that differences between the HBW and LBW groups may involve concerted remodeling of membrane lipid composition, rather than independent shifts in single lipid molecules.

Importantly, the interval between 110 and 130 d represented a notable shift in the lipidomic divergence between the HBW and LBW groups. This interpretation was supported by multiple lines of evidence from the lipidomic analyses. First, the predominant direction of PUFA-like differential lipids reversed between 110 and 130 d, shifting from HBW predominance at 90–110 d to LBW predominance at 130–150 d. Second, this directional reversal was driven largely by phospholipid classes, particularly PC and PE, indicating that the transition involved a reorganization of membrane lipid composition rather than a simple change in the total number of differential lipids. Third, the stage-related pattern analysis showed a broader redistribution from early TG-dominant differences toward later and persistent glycerophospholipid-dominant patterns. Taken together, these results indicate that the 110–130 d interval represents a developmentally relevant transition in lipid-related precursor composition. This transition is also biologically relevant to flavor precursor metabolism, because phospholipids carry oxidation-prone unsaturated fatty acyl chains that contribute to lipid-derived precursor pools during cooking. Similar age-dependent lipid remodeling has been reported in chicken breast muscle, supporting the view that membrane lipid organization is highly sensitive to developmental stage [30].

Overall, phospholipid remodeling represents the most prominent biochemical feature associated with differences between body weight-defined phenotypic groups in chicken breast muscle.

### 4.3. Body Weight-Defined Group Metabolic Differences Also Extend to Amino Acid- and Peptide-Related Precursor Pools

In addition to lipid-related molecules, several differential metabolites identified in this study were associated with amino acid and peptide metabolism, which may also be relevant to flavor precursor metabolism [2,3]. Among the metabolites consistently detected across stages were N-acetyl-L-methionine, N-methyl-L-glutamic acid, and γ-Glutamyl-5-hydroxytryptophan. These compounds are noteworthy because they may reflect broader alterations in sulfur amino acid-related, glutamate-related, and peptide-associated precursor metabolism. Moreover, N-acetyl-L-methionine and N-methyl-L-glutamic acid showed positive correlations with multiple differential lipids, implying that lipid- and water-soluble precursor pools may be remodeled in a partially coordinated manner rather than changing independently.

Rather than indicating direct flavor activity of each individual compound, these metabolites may reflect broader alterations in water-soluble precursor pools, including amino acid turnover, peptide metabolism, and related nitrogen metabolic processes. N-acetyl-L-methionine is a methionine-related metabolite and may reflect alterations in sulfur amino acid metabolism, which is relevant to flavor precursor chemistry because sulfur-containing amino acids contribute precursors for sulfur-containing volatiles formed during cooking [31]. N-methyl-L-glutamic acid may reflect altered glutamate-related metabolism, which is relevant because glutamate-associated compounds contribute to the umami-related chemical background of meat [32,33].

In addition, the peptide H-Ser-Leu-Ile-Gly-Arg-Leu-OH showed high VIP value at 90 and 150 d, and γ-Glutamyl-5-hydroxytryptophan exhibited clear stage-related variation, being up-regulated at 90 and 110 d but down-regulated at 130 and 150 d. Short peptides and γ-glutamyl compounds have been implicated in taste modulation, including kokumi-like and mouthfulness-associated sensory properties in meat and other food systems [33,34]. The positive correlations between several amino acid- or peptide-related metabolites and phospholipid-associated DLs further suggest that these two precursor pools may not vary independently, but instead may be co-regulated as part of broader metabolic remodeling during development.

Together, these observations suggest that metabolic divergence between body weight-defined phenotypic groups in BYC involves coordinated remodeling of both lipid-derived and water-soluble precursor pools. This coordinated remodeling may represent an important biochemical feature underlying differences in flavor precursor composition between HBW and LBW groups. Although the present study revealed coordinated developmental differences in lipid and metabolite profiles, the direct contribution of these molecules to sensory flavor traits was not evaluated and remains to be validated by targeted volatile, taste-active compound, and sensory analyses.

## 5. Conclusions

This study showed that differences between body weight-defined phenotypic groups in Beijing-You chicken breast muscle are stage-dependent rather than remaining consistent throughout growth. Across the four developmental stages, phospholipid remodeling emerged as the most dominant biochemical feature distinguishing HBW and LBW groups, as evidenced by the predominance of PC-, PE-, and PS-related differential lipids; the consistent enrichment of glycerophospholipid metabolism; and coordinated lipid-centered variation in the integrated analysis. Notably, the period from 110 to 130 d represented a key transition window, during which the direction of PUFA-like differential lipid abundance shifted from predominance in HBW birds to predominance in LBW birds, indicating a developmental reorganization of membrane lipid composition. In addition to lipid-related changes, metabolic differences between the HBW and LBW groups also extended to amino acid and peptide metabolites, suggesting that both lipid-derived and water-soluble precursor pools may contribute to flavor-related metabolic variation in chicken breast muscle. These findings provide new insight into the developmental metabolic basis of stage-dependent variation between body weight-defined phenotypic groups in flavor-related precursor composition, while the direct relationship between these precursor-level changes and flavor traits remains to be further validated.

## Data Availability

All relevant data supporting the findings of this study are provided in the manuscript and the supplementary information. Further raw data is available from the corresponding author upon reasonable request.

## Supplementary Materials

The following supporting information can be downloaded at: https://www.mdpi.com/article/10.3390/foods15091564/s1. Table S1: The body weight of the higher- and lower-body-weight groups at each developmental stage. Table S2: The differential lipids between HBW and LBW chickens for four age groups. Table S3: The differential metabolites between HBW and LBW chickens for four age groups. Figure S1: OPLS-DA score plots of lipidomic profiles. Figure S2: Permutation tests of lipidomic OPLS-DA models. Figure S3: OPLS-DA score plots and permutation tests of metabolomic profiles.

## Abbreviations

The following abbreviations are used in this manuscript:

HBW	Higher-body-weight
LBW	Lower-body-weight
BYCs	Beijing-You chickens
FAs	Fatty acyls
GLs	Glycerolipids
GPs	Glycerophospholipids
SPs	Sphingolipids
STs	Sterol lipids
PRs	Prenol lipids
SLs	Saccharolipids
PKs	Polyketides
OPLS-DA	Orthogonal projections to latent structures-discriminant analysis
VIP	Variable importance in projection
HMDB	Human Metabolome Database
TG	Triglyceride
PE	Phosphatidylethanolamine
PC	Phosphatidylcholine
dMePE	Dimethylphosphatidylethanolamine
PS	Phosphatidylserine
LPI	Lysophosphatidylinositol
MLCL	Monolysocardiolipin
SFAs	Saturated fatty acids
MUFAs	Monounsaturated fatty acids
PUFAs	Polyunsaturated fatty acids
