# Supplementary figures and images for: Integrated Lipidomics and Metabolomics Reveal Stage-Dependent Differences in Flavor Precursor Composition Between Higher- and Lower-Body-Weight Beijing-You Chickens
Source: Foods. 2026 May 1;15(9):1564. doi: 10.3390/foods15091564 (PMC13164383; doi:10.3390/foods15091564)

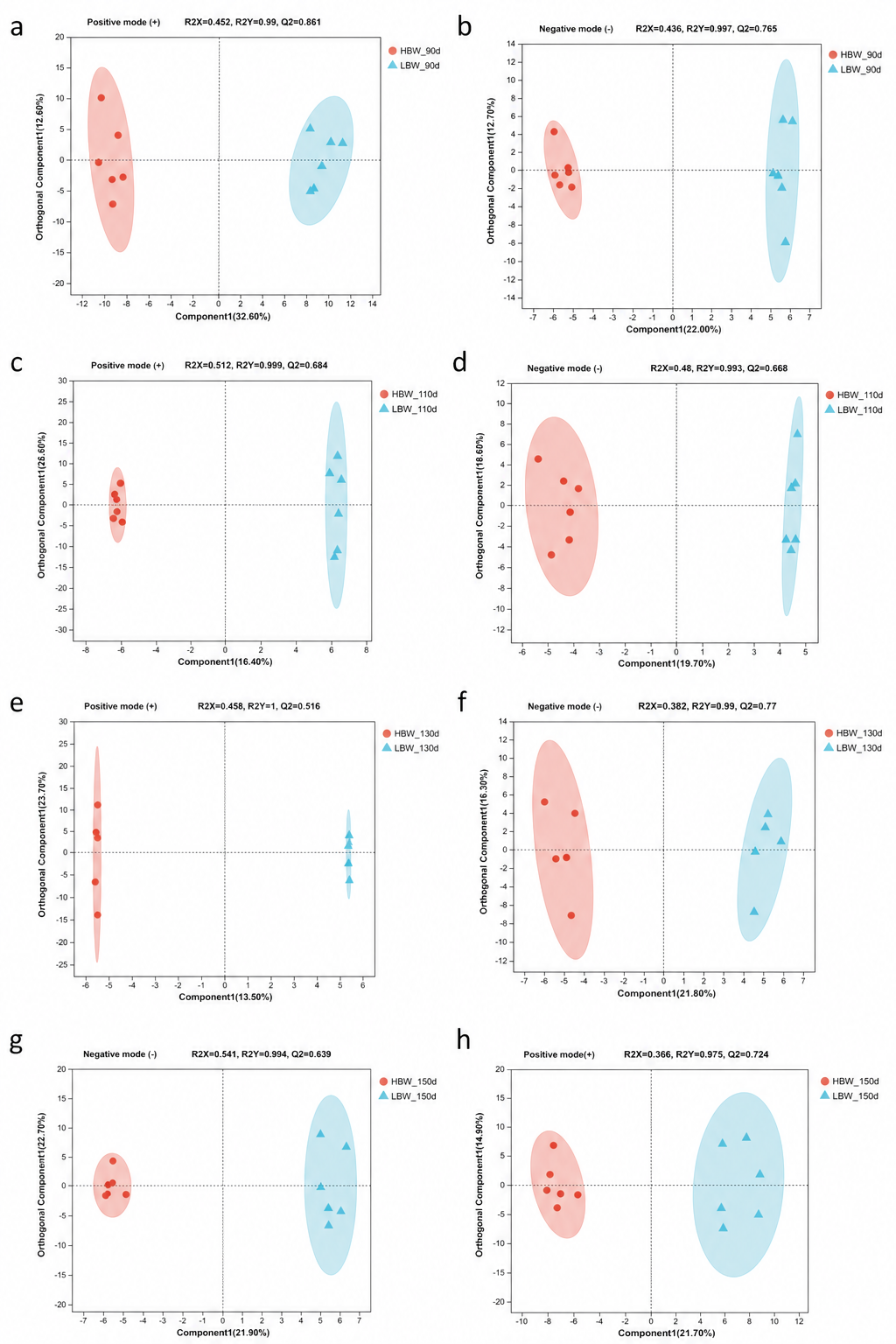

Supplement: Supplementary file 1 [file foods-15-01564-s001.zip › Figure S1.png]

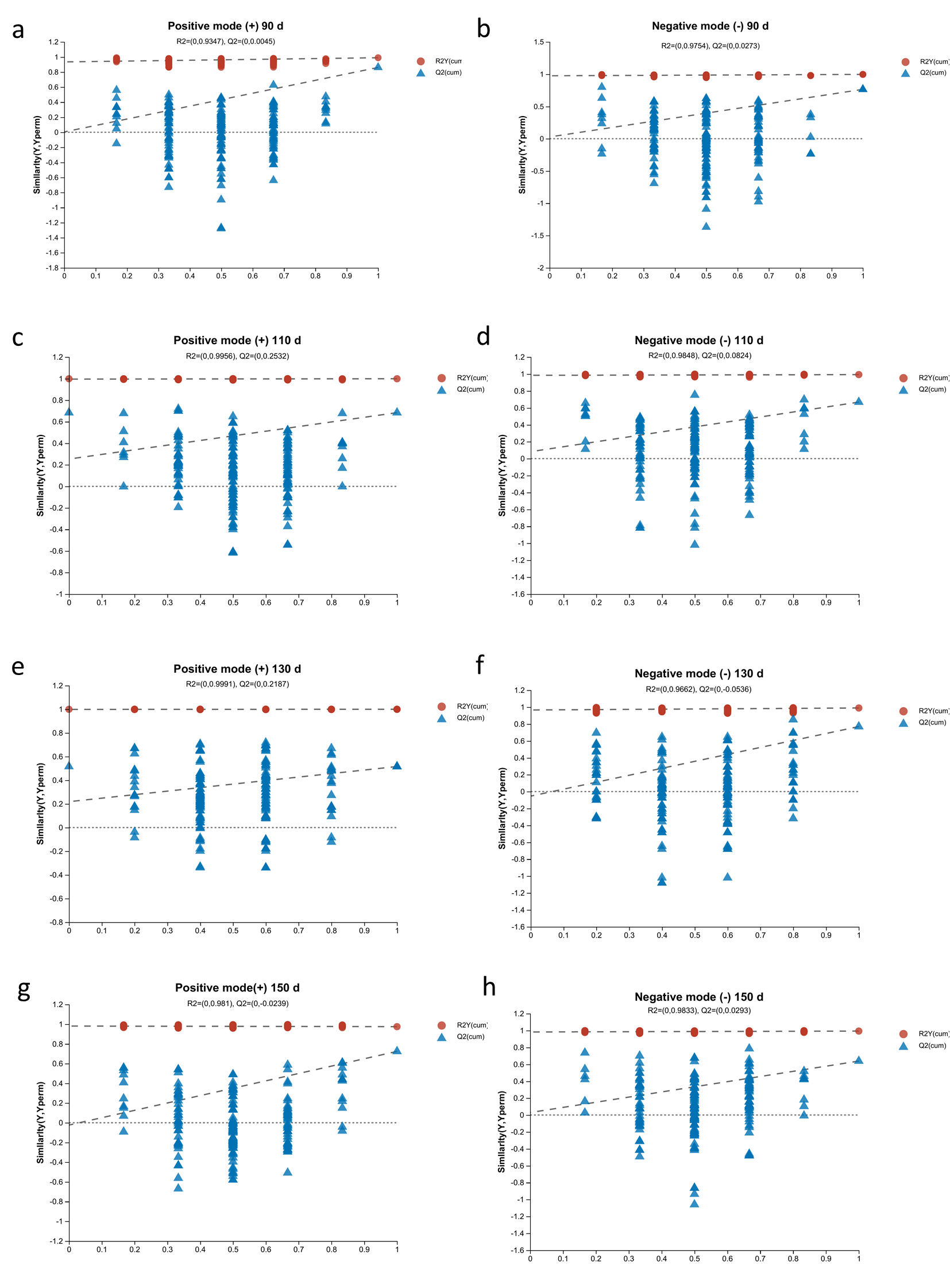

Supplement: Supplementary file 1 [file foods-15-01564-s001.zip › Figure S2.png]

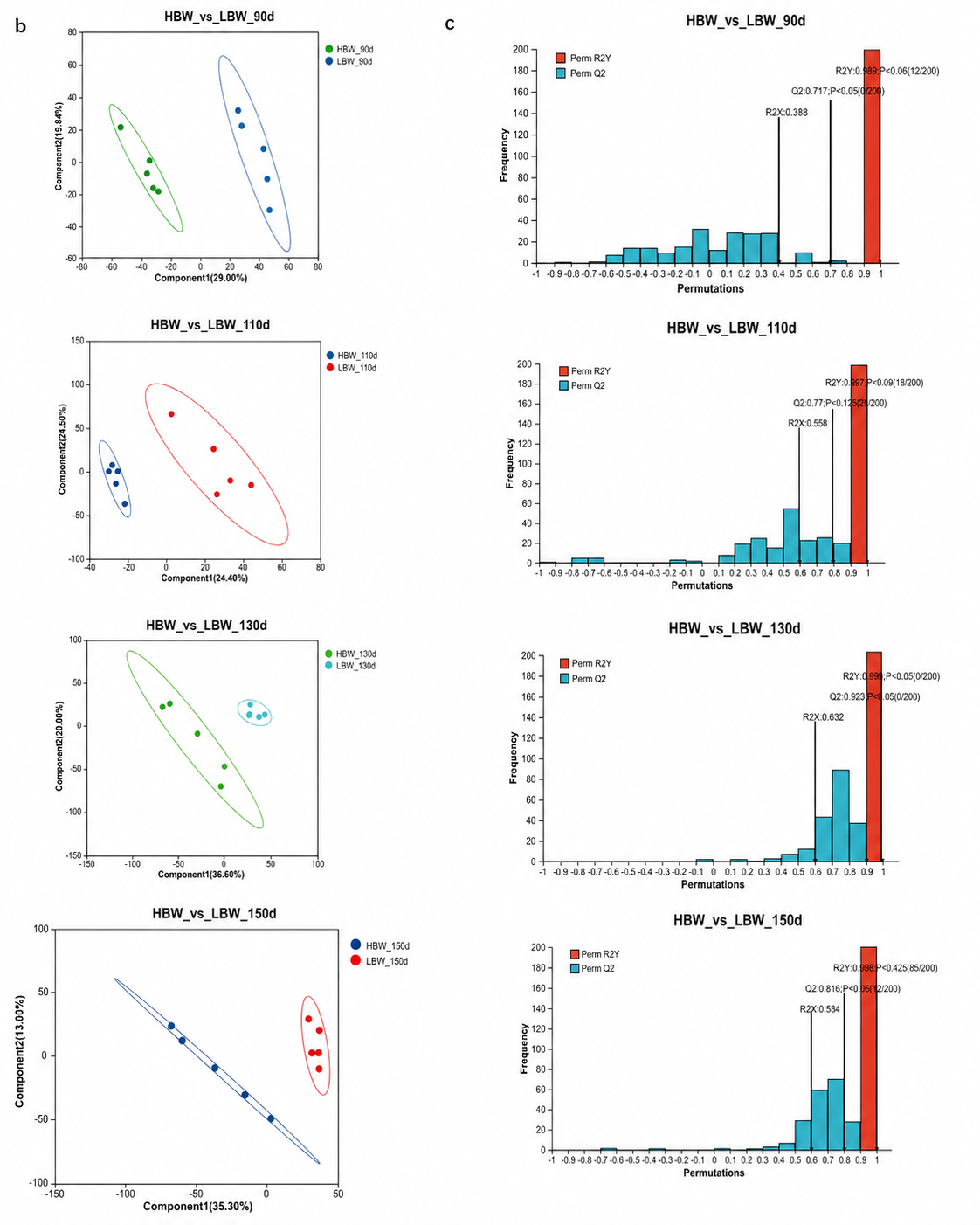

Supplement: Supplementary file 1 [file foods-15-01564-s001.zip › Figure S3.png]
